# Effect of lipemia and bilirubinemia on HIV-1 protease and reverse transcriptase genotyping and phenotyping success: a five-year analysis

**DOI:** 10.1186/1758-2652-13-S4-P148

**Published:** 2010-11-08

**Authors:** T Pattery, A Van Cauwenberge, M Van Hove, V Smits, K Van Soom, J Villacian

**Affiliations:** 1 Virco BVBA, Turnhoutseweg 30, Beerse, Belgium

## Background

Interference of clinical laboratory assays by endogenous & exogenous substances in the blood is well known. To date, there are no clear guidelines on the testing of lipemic or bilirubinemic (icteric) plasma in clinical laboratories. A conservative approach of alerting the physician with re-sampling and/or processing with caution is advised. This study focuses on the effect of processed (2005-2010) HIV-1 lipemic or icteric patient plasma (visual inspection) on genotyping (virco®TYPE HIV-1) and phenotyping (Antivirogram®) success at Virco.

## Materials and methods

The viral RNA extraction kits (QiaAmp Virus MDx kit: 965652 or Easymag Nuclisens: 280130-280135) are highly sensitive in deriving intact, good quality RNA from lipemic or icteric plasma, leading to successful amplification of PR- RT genes. The validity of the obtained result was confirmed by comparing the results from previous or subsequent visits/services from the same patient, where available.

## Results

Between 2005-2010, 569 lipemic (0.97% of total samples received) samples were processed. From the 510 genotype requests, 408 were successfully genotyped (positive & 265 had viral load (VL) >1000 cp/ml) and 102 failed (negative, 39 samples with VL <1000 cp/ml, 35 unknown VL & 28 with VL >1000 cp/ml). From the 335 phenotype requests, 267 were positive & 68 negative (36 with VL<1000 cp/ml, 16 unknown VL & 16 with VL >1000 cp/ml). From 2005-2010, 417 icteric (0.71% of total samples received) samples were processed. From the 394 genotype requests, 367 were positive (301 had VL >1000 cp/ml) & 27 negative (9 with VL<1000 cp/ml, 12 unknown VL & 6 with VL >1000 cp/ml). From the 166 phenotype requests, 153 were positive & 13 were negative (3 with VL<1000 cp/ml, 6 unknown VL & 4 with VL >1000 cp/ml).

## Conclusions

No limitations were observed for the different Clades. The success rate for lipemic samples (265/293) & icteric samples (301/307) with VL >1000 cp/ml was 90% & 98% respectively, indicating that both lipemic and icteric samples can be processed for resistance testing using our genotyping and phenotyping assays, under circumstances where re-sampling is difficult. The sensitivity to phenotyping clearly demonstrates the integrity of the amplified product that can be used to generate viable recombinant virus stocks.

